# ‘From Death, Lead Me to Immortality’ – Mantra of Ageing Skeletal Muscle

**DOI:** 10.2174/1389202911314040004

**Published:** 2013-06

**Authors:** Amarjit Saini, Sarabjit Mastana, Fiona Myers, Mark Peter Lewis

**Affiliations:** 1 School of Sport, Exercise and Health Sciences, Loughborough University, Loughborough, UK;; 2 School of Biological Sciences, Portsmouth University, Portsmouth, UK

**Keywords:** Epigenetics, Ageing, Satellite cells, Skeletal muscle, Methylation, Acetylation.

## Abstract

Skeletal muscle is a post-mitotic tissue maintained by repair and regeneration through a population of stem cell-like satellite cells. Following muscle injury, satellite cell proliferation is mediated by local signals ensuring sufficient progeny for tissue repair. Age–related changes in satellite cells as well as to the local and systemic environment potentially impact on the capacity of satellite cells to generate sufficient progeny in an ageing organism resulting in diminished regeneration. ‘Rejuvenation’ of satellite cell progeny and regenerative capacity by environmental stimuli effectors suggest that a subset of age-dependent satellite cell changes may be reversible. Epigenetic regulation of satellite stem cells that include DNA methylation and histone modifications which regulate gene expression are potential mechanisms for such reversible changes and have been shown to control organismal longevity. The area of health and ageing that is likely to benefit soonest from advances in the biology of adult stem cells is the emerging field of regenerative medicine. Further studies are needed to elucidate the mechanisms by which epigenetic modifications regulate satellite stem cell function and will require an increased understanding of stem-cell biology, the environment of the aged tissue and the interaction between the two.

## INTRODUCTION

The effects of ageing have serious physiological consequences on skeletal muscle. The term ‘Sarcopenia’ has been used to describe the slow, progressive loss of muscle mass with advancing age [1]. Operationally, sarcopenia is defined as an appendicular skeletal muscle mass divided by height in metres of more than two standard deviations below the young normal mean. Associated with this condition is the gradual decline in muscle functional properties, including a decrease in force producing capacity and maximum velocity of shortening and a general slowing of contraction and relaxation [1].

**Fig. (1):**
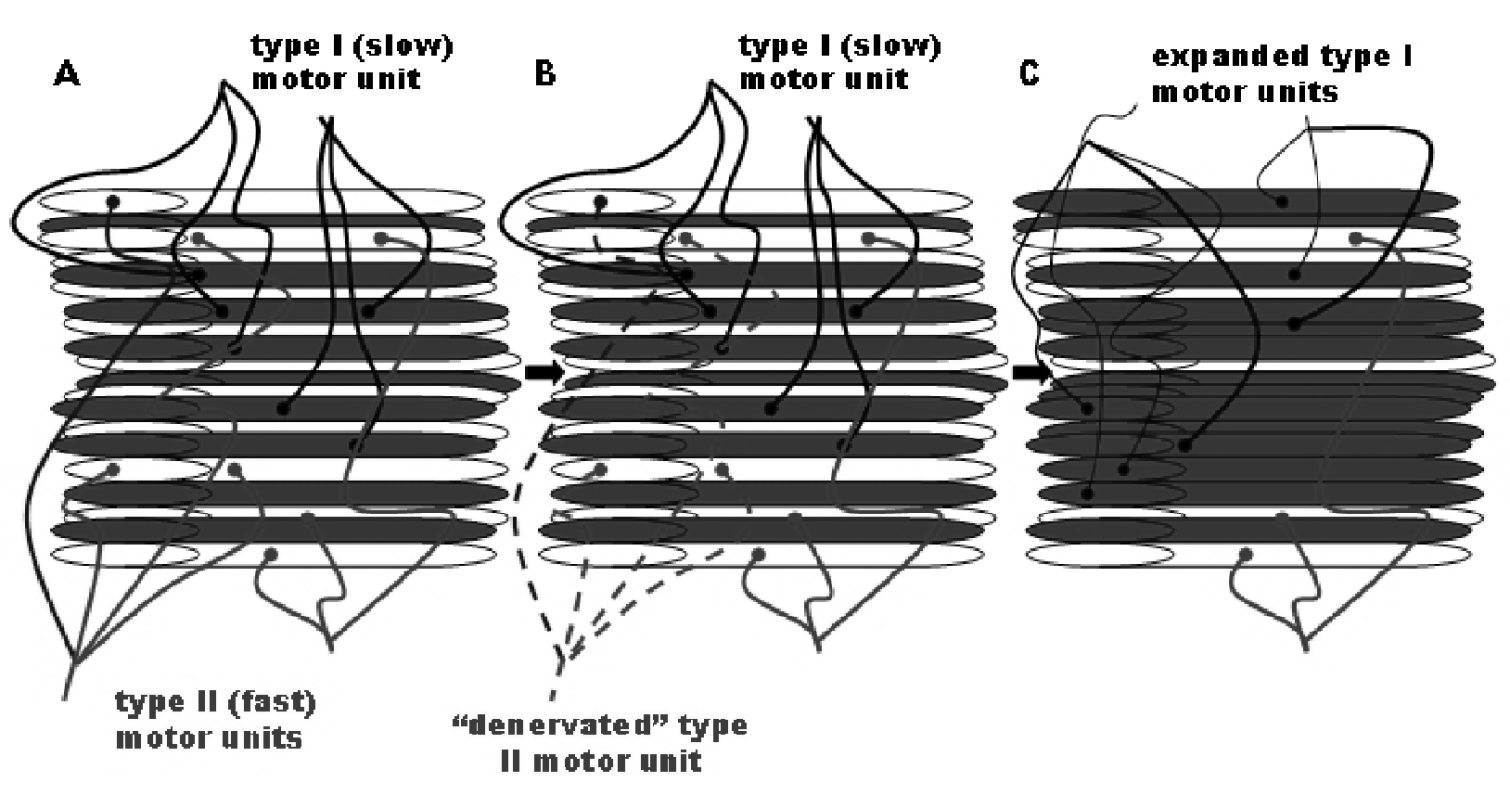
Age-related motor unit remodelling [2]. The process of muscle fibre denervation and reinnervation is believed to be ongoing throughout life and is accelerated with ageing [2, 85]. As ageing progresses there is believed to be a selective loss of fast, type II motor units, with denervated fibres either being lost or reinnervated by slow, type I motor units. White fibres are those that have been denervated recently and which will either die or be reinnervated by another (type I) motor neuron. The CSA of remaining fibres is also thought to decrease. Thus, muscles become smaller (due to overall loss of fibres) and slower (due to increased proportion of type I fibres) with increasing age [1, 2]).

The age-related decline in muscle function is believed to be related to a decrease in both muscle quantity (mass) and a decrease in muscle quality (a term encompassing many factors including strength per muscle cross-sectional area (CSA), fibre proportions and metabolic characteristics). The loss of muscle quantity occurs through a decrease in contractile protein content via the loss of individual muscle fibres and a decrease in the size of remaining muscle fibres (Fig. **1**) [2]. These changes affect the different muscle fibre types selectively, a process termed ‘age-related motor-unit remodelling (Fig. **1**) [3]. In addition to the remodelling of the aged muscle towards a slower phenotype, CSA is reduced significantly in type II (fast) fibres, whereas type I (slow) fibres remain relatively unaffected [4]. Similarly, it is mostly the type II fibres that are lost with ageing, leaving a higher proportion of type I fibres in the muscle [5].

Various mechanisms have been put forward to explain the change in total muscle mass (see Table **1**). Proposed mechanisms generally include a lack of regular physical activity (sedentary lifestyle), alterations in endocrine function (insulin, testosterone, growth hormone, cortisol), a loss of neuromuscular function (denervation or reinnervation), a change in protein metabolism (a deficit between protein synthesis versus degradation), nutrition (primarily amino acids), apoptosis and disease or trauma [2, 6].

With advancing age (>85 years) loss of mass and strength can have a significant impact on functional independence [7]. Data from the Established Populations for Epidemiologic Studies of the Elderly (EPESE) show even a loss of 10% or more of total body weight between the age 50 and 67 years is associated with a 60% increase in mortality compared to persons with stable weight [8]. In institutionalised nursing home residents, a 10% loss of total body weight over a 6-month interval strongly predicted mortality in the ensuing 6 months [9]. In residents who lost at least 5% of their body weight, a 5- to 10-fold increased risk of death was reported [10]. The age-related loss of muscle mass appears to be reasonably constant, at a rate approximately 1–2% per year over the age of 50 years [11]. This decline in muscle mass occurs in both sedentary and active ageing adults [6]. In contrast, there is no net change in rates of protein synthesis and degradation in healthy young adults. Whilst the effects of ageing on skeletal muscle are inevitable, it is unclear whether these intrinsic changes are immutable or reversible.

## SKELETAL MUSCLE REPAIR

**Fig. (2):**
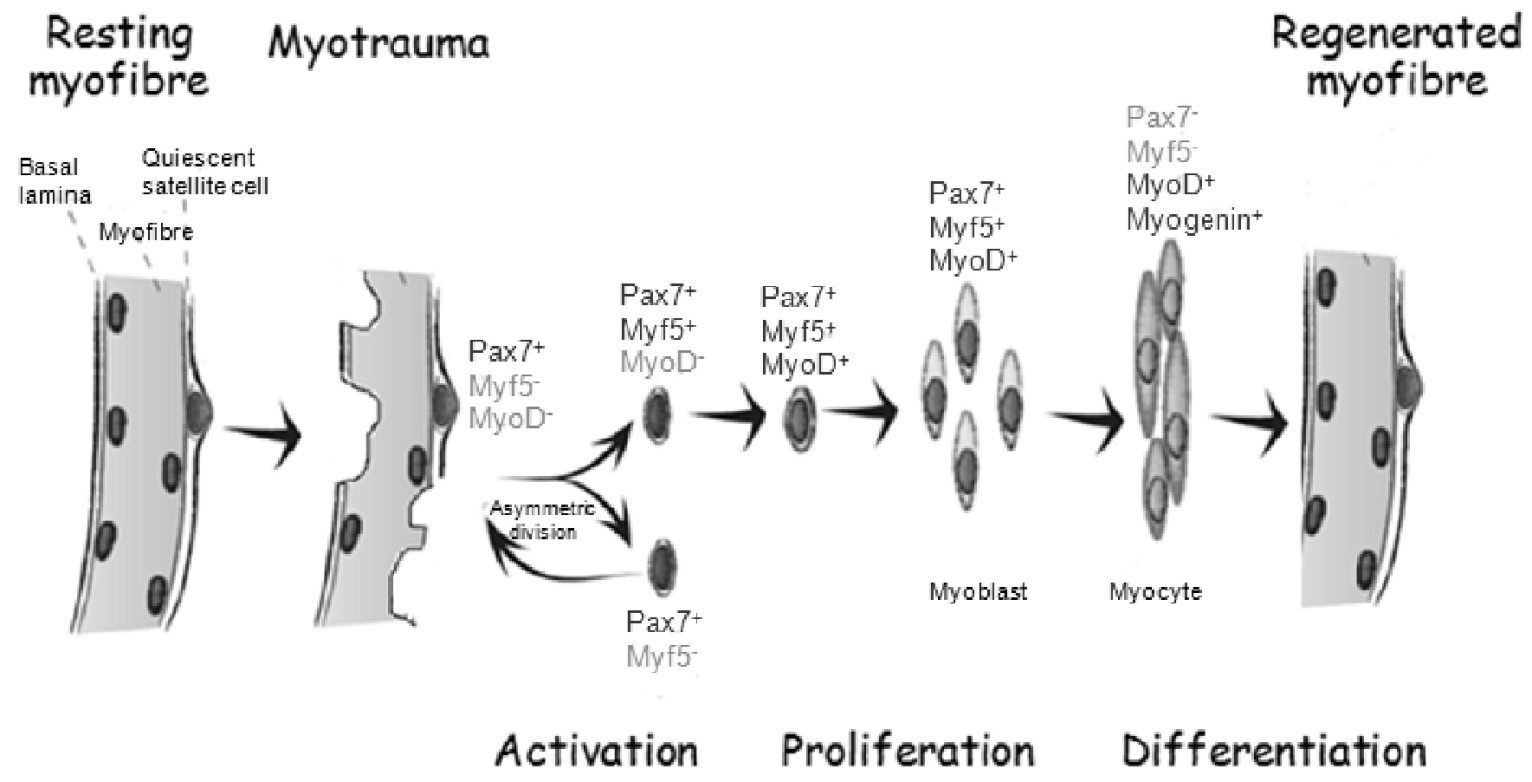
Satellite cell response to myotrauma (adapted from [86]). Quiescent satellite cells in adult muscles are characterised by Pax7 expression. Upon injury or workload, satellite cells are activated and divide asymmetrically, thereby generating a self-renewing cell and a committed progenitor which begins to express the muscle regulatory factor Myf5. Myf5-expressing cells enter the cell cycle, undergo rounds of proliferation turning into MyoD-expressing myoblasts, which later on will express Myogenin and downregulate Pax7. This pool of cells will differentiate and fuse to form new myofibers during adult muscle regeneration.

Skeletal muscle is composed predominantly of post-mitotic, multi-nucleated muscle fibres, which account for up to half of the mass of the human body. Lifelong maintenance of skeletal muscle function depends in large part on preserving the regenerative capacity of muscle fibres, which may be subjected to a variety of physical and biochemical insults that introduce substantial muscle damage. Thus, successful maintenance of muscle function throughout life represents a critical and important challenge to maintaining a healthy and active lifestyle. Repair of injured fibres requires a unique population of tissue-specific muscle stem cells called satellite cells [12, 13]. These cells play a crucial role in regeneration of myofibres responding to environmental stimuli following injury or transplantation in humans and mice [14-19]. They reside beneath the basal lamina of mature muscle fibres, maintaining a close physical interaction that enables an exquisite sensitivity to muscle injury [12]. It is essential that satellite stem cells over the course of a lifespan maintain an undifferentiated, yet committed state, primed to respond to the environment. In the process of self-renewal, satellite cells spend most of their lifetime in a quiescent state, upon muscle damage they can re-enter the cell cycle and either: undergo a symmetric cell division to self-renew and expand the satellite cell population; or undergo an asymmetric cell division that results in the cell on the basal lamina side maintaining the satellite cell identity, while the cell adjacent to the muscle fibre enters the myogenic differentiation programme (Fig. **2**) [20-22].

Skeletal muscle injury results in an influx of inflammatory cells that remove necrotic debris from the tissue. Following the inflammatory response, Pax7-expressing satellite cells enter mitosis to generate progeny by undergoing repeated rounds of proliferation. These cells migrate to the site of damage and a high proportion of this progeny undergo myogenic differentiation restoring the damaged muscle fibres, whilst others undergo self-renewal to repopulate the muscle as satellite stem cells [23]. Upon activation by muscle injury, proliferating satellite cells become myoblasts through upregulation of a myogenic regulatory factor (MRF) called myoblast determination protein (MyoD) [24]. MyoD drives proliferation by controlling re-entry into the cell cycle and activates the transcription of muscle-specific genes [25]. As a last step, downregulation of paired box 7 (Pax7) and upregulation of myogenin prime the myoblasts to become myocytes. These cells are terminally committed, exit the cell cycle and fuse with other myoblasts or existing fibres. This process will finally repair the damaged muscle tissue (Fig. **2**) [26]. Satellite cell self-renewal and the transcription factors controlling lineage progression during regeneration are thought to be regulated by a variety of extrinsic cues [27]. Satellite cells are thus critical for tissue regeneration, response to injury and tissue plasticity in adult skeletal muscle and meet the functional definition of what stem cells are, as they have the ability to self-renew, in addition to producing differentiating progeny [28]. Considering the key role of satellite cells in myofibre repair, compromised satellite cell function has been proposed to contribute to age-linked muscle deterioration [29]. Whether satellite cell potential for self-renewal and the capacity to properly differentiate progressively diminishes during ageing, and if this is the case, whether these defects are entirely irreversible, requires further investigation.

## EPIGENETIC CHANGES: A PIVOTAL MECHANISM FOR SATELLITE CELL REGULATION IN SKELETAL MUSCLE REGENERATION DURING AGING?

Age-related changes to stem cells and their environment may be categorised into two groups: irreversible versus reversible (Table **2**) [30]. Irreversible damages to ageing stem cells include intrinsic changes may be associated with nuclear/mitochondrial DNA damage and telomere shortening, which have previously been reviewed in detail [30-35]. In contrast, changes to the systemic and local signalling environment that also occur during ageing may be reversible. 

It is proposed that the age-related changes to adult stem cells, such as muscle satellite cells, are strongly influenced by environmental factors during ageing. As discussed above, lack of regular physical activity (sedentary lifestyle), alterations in endocrine function (insulin, testosterone, growth hormone, cortisol)/growth factor signalling, a loss of neuromuscular function (denervation or reinnervation), a change in protein metabolism (a deficit between protein synthesis versus degradation), nutrition (primarily amino acids), apoptosis and disease or trauma (Table **1**) [2, 6] may impact on skeletal muscle and capacity of satellite cell activation suggesting that age-dependent changes may be reversible in these tissues and diminished regenerative capacity of ageing skeletal muscle may be the result of the aged skeletal environment on satellite cell function and not primarily intrinsic ageing.

Consequently, if environmental cues indeed play a pivotal role in stem cell function it is worthwhile to consider the mechanisms by which epigenetic modification could mediate these responses. ‘Epigenetics’, in the strict definition of the term, is the study of phenotypic or gene expression patterns heritable through cell division that are independent of DNA sequence [36]. More recently a broader definition of epigenetics as the dynamic regulation of gene expression by sequence-independent mechanisms has been proposed, which include changes in DNA methylation and histone modifications [37-39]. In the next section of this review, we discuss epigenetic regulation of satellite stem cells with specific focus on the regulation of chromatin state by chromatin-modifying complexes and the role of DNA methylation that are pivotal for satellite stem cell proliferation and fusion. We propose that epigenetic mechanisms could co-ordinately control adult stem cell gene expression programmes during ageing and that integrated environmental stimuli to trigger changes to stem cells through chromatin and DNA methylation may be reversible and thus ideally situated to be molecular effectors of satellite cell ‘rejuvenation’ and muscle regeneration.

## CHROMATIN REMODELLING IN SATELLITE CELLS

**Fig. (3):**
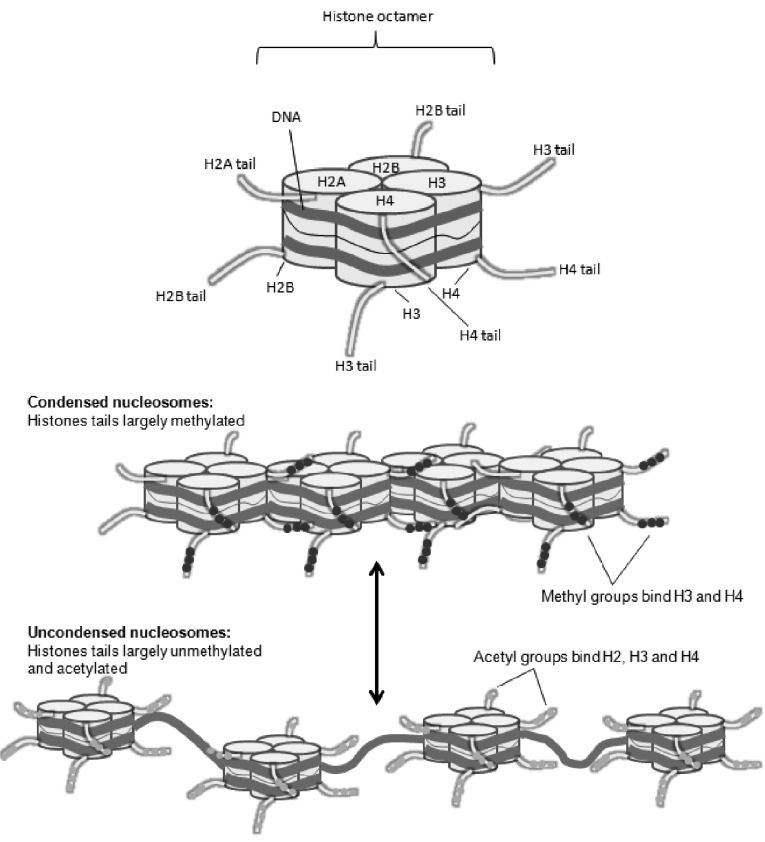
Arrangement of the nucleosome. Histone tails protruding from the nucleosome subunits allow for the attachment of chemical groups. Methyl groups condense nucleosomes more tightly preventing gene transcription. Acetylation loosens nucleosome packing, exposing the DNA to RNA polymerase and transcription factors that will activate genes.

In defining the network of transcription factors involved in satellite cell activation, proliferation and differentiation it has become apparent that various elements of the epigenetic and chromatin machinery participate. At a molecular level, DNA is wrapped around a nucleosome consisting of a core of four histone proteins (histone 2A, 2B, 3 and 4) (Fig. **3**) to form chromatin, which not only plays a role in chromosomal stability, but also regulates the expression of surrounding genes [40]. The mechanisms of histone regulated gene expression can be attributed to post translational modifications to histone tails [40]. These modifications, or ‘epigenetic marks’, include methylation, acetylation, sumoylation and phosphorylation of amino acid residues within histone tails. The type of mark and the amino acid residue that is tagged are key determinants of either transcriptional activation or repression [40].

Changes in chromatin structure are necessary not only to access target sequences but also to endow local chromatin with specific states of transcriptional competence. Repression and activation are controlled to a large extent by modifying the tails of histones H3 and H4 by two small organic groups: methyl (CH_3_) and acetyl (COCH_3_) residues. In general, histone acetylation – the addition of negatively charged acetyl groups to histones – neutralises the basic charge of lysine and loosens the histone. This activates transcription. There are two main enzymatic activities that modify chromatin and regulate access to DNA: chromatin-modifying complexes and chromatin-remodelling complexes [41, 42]. Chromatin-modifying complexes contain the subunits of different histone-modifying enzymes that catalyse reversible post-translational modification of histones and other factors [43]. Enzymes known as histone acetyltransferases place acetyl groups on histones (especially on lysine in H3 and H4), destabilising the nucleosomes so that they come apart easily. In contrast, enzymes that remove acetyl groups – histone deacetylases – stabilise the nucleosome and prevent transcription. Histone methylation, the addition of methyl groups to histone methyltransferases, can either activate or further repress transcription, depending on the amino acid being methylated and the presence of other methyl or acetyl groups nearby [40] Histone modifications such as acetylation of various lysine residues of histones H3 and H4 and trimethylation of lysine 4 of histone H3 (H3K4me3) are both associated with active gene expression. Diversely trimethylation of lysine 27 of histone H3 (H3K27me3) is implicated in genomic regions enriched for both active K4 and repressive K27 histone mark and is thought to keep genes silent but poised for activation [44].

Studies in human and mouse embryonic stem cells have identified histone methyltransferases involved in depositing the transcriptionally repressive mark trimethyl histone H3 at lysine 27 (H3K27me3) on developmentally regulated genes and transcriptionally permissive mark trimethylation of H3 at lysine 4 (H3K4me3) and these potentially mediate satellite cell activation. Upon muscle injury, satellite cells express Pax7 become activated and re-enter the cell cycle and begin to express cell-cycle regulatory genes, which become marked by H3K4me3 [45]. Satellite cells proliferation to skeletal myoblasts is characterised by the expression of Myf5/MyoD and coincides with enrichment of the transcriptionally permissive H3K4me3 mark within the chromatin [46]. Subsequent, formation of multinucleated myotubes requires the downregulation of Myf5, and cell-cycle regulatory genes, and the activation of myogenin. Expression of the myogenin gene coincides with the removal of the repressive H3K27me3 mark [47, 48] and the appearance of the transcriptionally permissive H3K4me3 mark within the 5’end of the gene [48, 49]. Coincident with the terminal differentiation, myoblasts exit the cell cycle as regulators of this process are silenced through incorporation of the H3K27me3 modification into chromatin marking their respective genes [50]. These studies demonstrate epigenetic marking of chromatin in proliferating and differentiating myoblasts. However studies are currently restricted to a limited number of genes, advances in high-throughput sequencing should soon provide the epigenetic status for the entire muscle transcriptome at different stages of muscle regeneration.

The combination or sequential addition of various post-translational modifications to the terminal amino acid residues of histones has different functional consequences for gene activity and chromatin organisation, and they are thought to form a histone code [40]. The modification of a specific residue is maintained through the activity of various enzymes. For instance, the acetylation status of histones is maintained by the balance between histone acetyltransferases (HATs) and histone deacetylases (HDACs). HATs are associated with acetylating certain transcription factors, thereby influencing their activity [41], conversely, histone deacetylation is generally associated with transcriptional repression. In a recent genome wide study of MyoD binding during myogenesis, it has been demonstrated that MyoD associates with histone acetylation in both myoblasts and myotubes, indicating that on top of regulating the muscle gene programme through its transcriptional activity, MyoD could also coordinate the acetylation status of myogenic genes [51]. Studies have shown altering expression of mammalian Sirt1 protein deacetylase homologue of the yeast Sir2, to be associated with chromatin remodelling playing a pivotal role in gene silencing and in prolonging life. Extensively investigated in yeast, Sir2 is an NAD-dependent protein deacetylase [52] with a role in transcriptional silencing at the ribosomal RNA, telomere and mating type loci [53]. In cell culture models, our studies in C2C12 skeletal myoblasts in which chronic low level cytokine, Tumour Necrosis Factor (TNF-α), administration to artificially mimic systemic inflammation in ageing resulted in a decline in Sirt1 mRNA expression. This was accompanied by increased apoptosis, decreased proliferation and the capacity of the myoblasts to form myotubes blocked [54, 55]. This suggested that the environmental conditions (chronic cytokine environment) was able to impact on the myoblast epigenome by altering Sirt1 activity and subsequent histone modification inducing transcriptional repression of genes associated with differentiation. In a post-mitotic skeletal muscle this would impact on satellite cell capacity to regenerate or repair damaged myofibres. Interestingly, the potential plasticity of the myoblast epigenome was demonstrated when resveratrol which acted to increase expression of Sirt1 was able to ameliorate the negative effects of chronic low grade cytokine stimulation highlighting the potential use of pharmacological agents to alter satellite cell epigenome to mediate regeneration. Overall these findings indicate that well-regulated maintenance of chromatin—and thereby access to genes controlling self-renewal, differentiation, cellular metabolism, DNA damage repair is likely to be critical for maintaining the functional capacity of adult satellite cells. Moreover, studies indicate changes to the epigenetic marking of chromatin are thought to be reversible therefore are ideally situated to be molecular effectors of satellite cell rejuvenation. 

## DNA METHYLATION IN SATELLITE CELLS

**Fig. (4):**
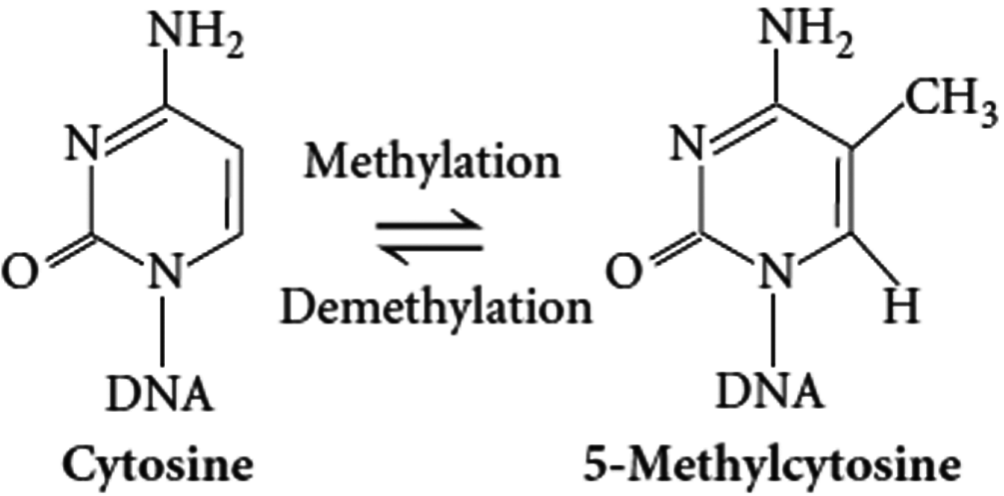
Methylation of cytosine to 5-methylcytosine.

In addition to the posttranslational modification of histones, CpG dinucleotide methylation is a major source of epigenetic information and the methylation of CpG containing promoters represses the expression of specific genes. DNA methylation preferentially occurs at the C5 position of cytosine (Fig. **4**) in the context of CG, forming the minor bases, 5-methylcytosines, which account for approximately 1% in the mammalian genome [56, 57].

**Fig. (5):**
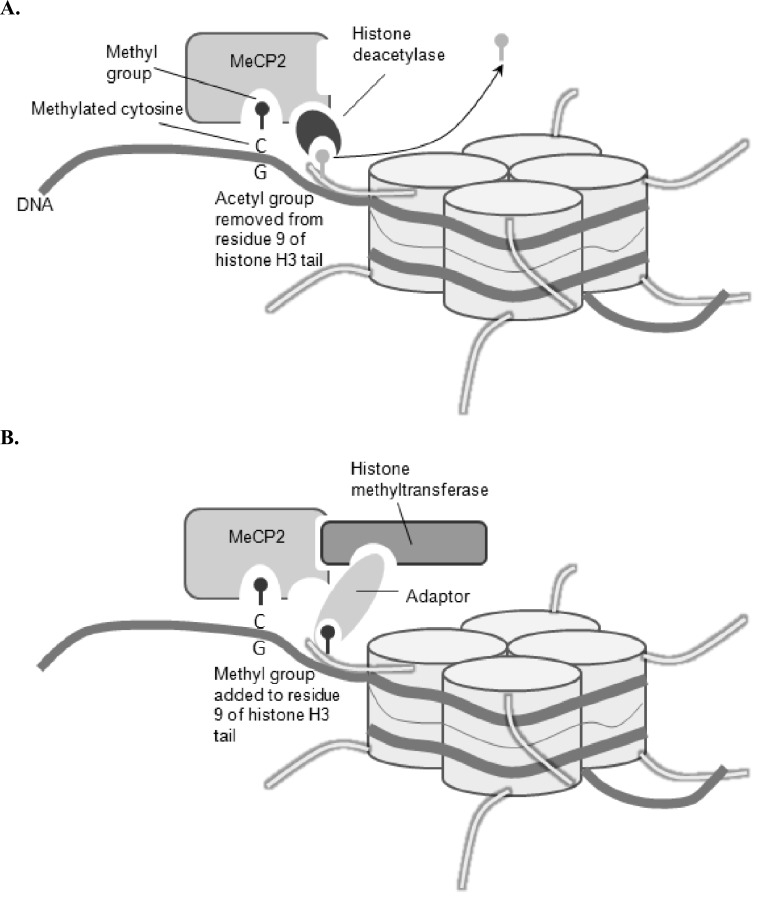
Modifying nucleosomes through methylated DNA. MeCP2 recognises the methylated cytosines of DNA. It binds to the DNA and thereby is able to recruit histone deacetylases (which take acetyl groups off the histones) (**A**) or histone methyltransferases (wich add methyl groups to the histones) (**B**). Both modifications promote the stability of the nucleosome and the tight packing of DNA, thereby repressing gene expression in these regions of DNA methylation [87, 88].

**Fig. (6):**
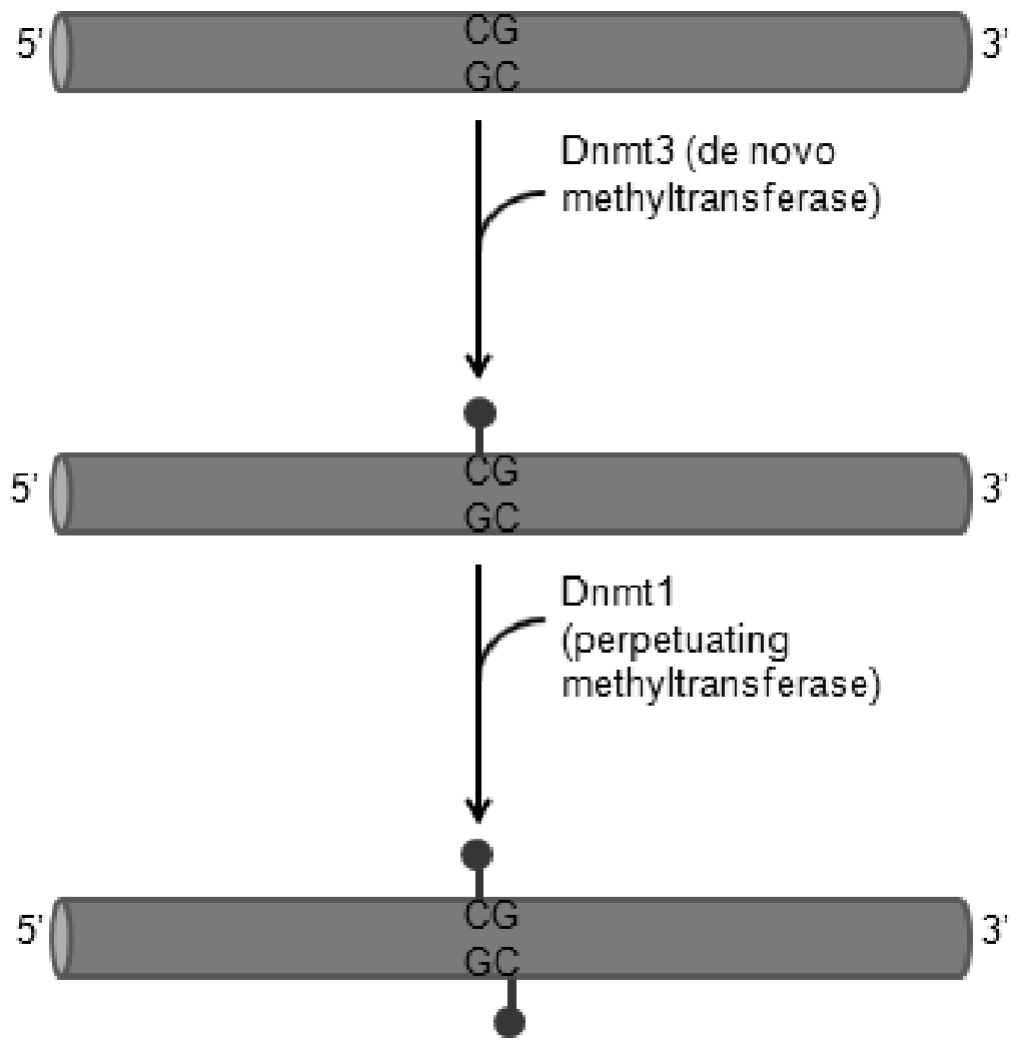
Two methyltransferases are critical in modifying DNA (adapted from [58]). The ‘*de novo*’ methyltransferase Dnmt3 can place a methyl group on unmethylated cytosines. The ‘perpetuating’ methyltransferase, Dnmt1, recognises methylated Cs on one strand and methylates the C on the CG pair on the opposite strand.

In eukaryotes, the presence of methylated cytosines in a gene’s promoter correlates with the repression of transcription from that gene. DNA methylation appears to act in two ways to repress gene expression. First, it can block the binding of transcription factors to enhancers. Several transcription factors can bind to a particular sequence of unmethylated DNA but cannot bind to that DNA if one of its cytosines is methylated. Secondly, a methylated cytosine can recruit the binding of proteins that facilitate the methylation or deacetylation of histones, thereby stabilising the nucleosomes. For instance, methylated cytosines in DNA can bind particular proteins such as methyl-CpG-binding proteins (MeCPs) that can bind to histone deacetylases and histone methyltransferases, which respectively, remove acetyl groups and add methyl groups on the histones (Fig. **5**). As a result, the nucleosomes form tight complexes with the DNA preventing other transcription factors and RNA polymerases complexing with the genes (Fig. **5**) [58]. Other enzymes recruited to the chromatin by MeCPs are DNA methyltransferases (DNMTs) [56, 57]. DNA methyltransferase-3 (Dnmt3) for instance is able methylate previously unmethylated cytosines on the DNA increasing the region that can be repressed (Fig. **6**). The newly established methylation pattern can then be transmitted to the next generation by DNA methyltransferase-1 (Dnmt1). This enzyme recognises methyl cytosines on one strand of DNA and places methyl groups on the newly synthesised strand opposite it (Fig. **6**). Thus, in each cell division the newly synthesised (unmethylated) strand will become properly methylated when Dnmt1 binds to the methyl on the old CpG sequence and methylates the cytosine of the CpG sequence on the complementary strand. In this way, when the DNA methylation pattern is established in a cell, it can be stably inherited by all the progeny of that cell [58].

Oikawa *et al. *[59] investigated the role of methyl-CpG-binding protein, CIBZ, in muscle differentiation through regulation of myogenin. CIBZ (CtBP-interacting BTB zinc finger protein, ZBTB38) possesses a modified zinc finger domain capable of binding methylated DNA *in vitro *and can act as a transcriptional repressor of methylated reporter genes [60]. Following muscle damage, satellite cells re-enter the cell cycle and proliferate as myoblasts to repopulate the satellite cell pool and undergo myogenic differentiation. Myogenin is activated early in this differentiation process and is one of the key transcription factors involved in coordinating myogenic differentiation. The myogenin promoter in developing somites is initially methylated prior to activation and is subsequently demethylated [61]. Oikawa *et al. *[59] showed in the myoblast cell line C2C12s expression of CIBZ and myogenin proteins are reciprocally regulated when satellite cells were induced to differentiate. Thus, CIBZ is required to suppress myogenic differentiation in satellite cells *in vitro*, most likely due to its role in the transcriptional repression of the key myogenic differentiation factor myogenin. These findings provide evidence in muscle for a network between DNA methyltransferases inducing methylation at the 5′-position of cytosine (in CpG dinucleotides) and methyl binding proteins associated with transcriptional repression and implicated in maintaining patterns of gene expression during differentiation.

Studies have shown, DNA methylation is thought to repress many muscle gene loci and DNA methyltransferase inhibitors, such as 5-azacytidine can induce the transdifferentiation of fibroblasts into myoblasts [62, 63]. This effect may be mediated by the repression of the MyoD promoter through demethylation (reviewed by Palacios and Puri, [64]) and demethylation of the myogenin promoter, since both events appear to be necessary for differentiation to proceed [64, 65]. Recent studies have demonstrated that DNA methylation helps to restrict myogenin activation until both MEF2A and SIX1 transcription factor are coexpressed, both during embryonic myogenesis and in myoblasts. Interestingly, a comprehensive analysis of CpG and non-CpG moieties in the myogenin promoter showed that expression of the gene and, consequently, terminal differentiation, are linked to the methylation starting levels and the kinetics of demethylation of some specific clusters of C situated outside of CpG islands [66].

As above studies have demonstrated the role of the epigenome in myoblast differentiation we also have studied DNA methylation/demethylation kinetics in transcriptional regulation of myogenin and myotube formation using C2C12s to investigate the affects of cellular ageing on satellite cell function in regeneration/repair [67]. We have previously investigated the impact of cellular divisions on subsequent myoblast behaviour in C2C12s to mimic satellite cell division and renewal [67]. Adopting multiple population doublings model to investigate the impact of serial divisions on muscle cell behaviour focusing on the transition from proliferation to differentiation we reported that C2C12 cells that have undergone 58 population doublings show inappropriate cell cycle progression with reduced differentiation and associated changes in key molecular pathways IGF-I/Akt and myogenin expression [68]. The model presents similarities with *in vivo* human muscle and isolated muscle cells from elderly individuals, where reduced differentiation of satellite cells and regenerative capacity is observed. Our studies have shown C2C12s undergone multiple passages as a model of ageing satellite cells myogenin gene becomes highly methylated resulting in transcriptional repression thus attenuating the capacity of these myoblasts to differentiate in comparison to myoblasts that have undergone fewer population doublings (unpublished). The subsequent use of a methyltransferase inhibitor 5-azacytidiine (5-AZ) to lower levels of DNA methylation in late passage C2C12s was able to stimulate fusion capacity to similar levels observed in myoblasts that had undergone fewer population-doublings (unpublished). These findings have implications with regards to how DNA methytlation of the epigenome may influence transcriptional repression of satellite cells with ageing and their capacity to be recruited in muscle regeneration. The study also provides evidence that old stem cells can be ‘rejuvenated’ in this circumstance using a pharmaceutical agent. This raises the possibility that a subset of age-dependent stem cell changes is regulated by reversible mechanisms. Epigenetic regulators may therefore be good candidates to mediate plastic changes in gene expression. 

## EPIGENETIC MODIFICATIONS? SATELLITE CELLS AND THE AGEING SKELETAL MUSCLE ENVIRONMENT

It is proposed impaired ability to produce Mechano Growth Factor (MGF a specific splice variant of IGF-I) subsequently leading to epigenetic modifications in satellite cell progeny may explain why older populations experience increased loss of muscle mass. The consequent decline in satellite cell number, which provides additional myonuclei to repair damaged myofibres or for increased rates of protein synthesis to facilitate hypertrophy, could explain why older individuals take longer to gain an equivalent percentage increase in muscle mass compared to young participants following a single bout of resistance exercise. A study conducted by Hameed and co-workers [69] in which young (25–36 years) and older (70–82 years) males were subjected to resistance exercise supports this hypothesis, and demonstrates that MGF mRNA was significantly increased in the muscle of the young but not the older participants. In addition, Hameed and colleagues reported an approximate two-fold increase in MyoD expression in the younger group, and a slight but non-significant decline in its expression in the older group. Thus pointing to the suggestion that satellite cell activity (myoD expression) is upregulated in the young, allowing for faster recovery following a bout of exercise, compared to inhibition of their activity in the older group.

**Fig. (7):**
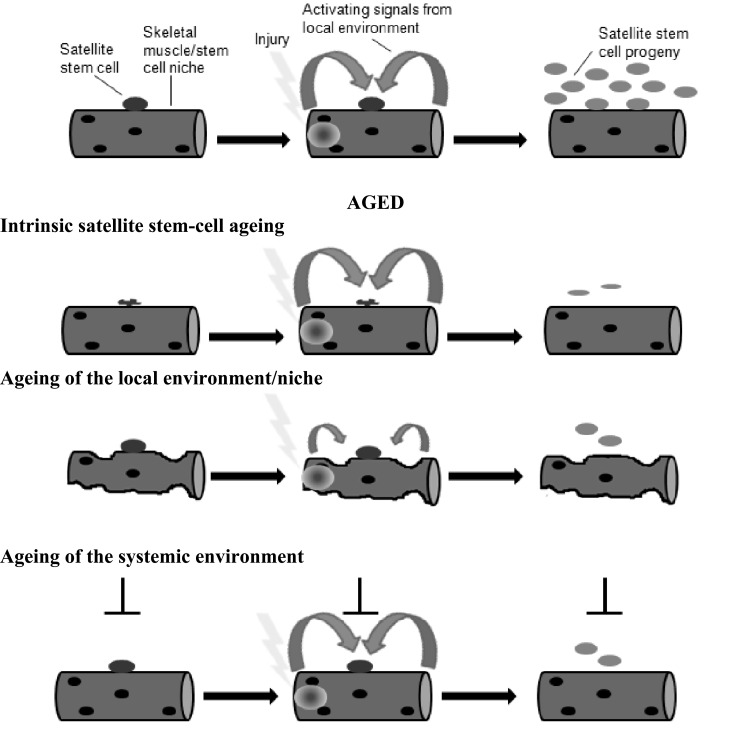
Ageing of stem-cell functionality (adapted from [89]). The decline in stem cell functionality with age can be due to age-related changes at many levels. This figure illustrates several possibilities using a skeletal muscle fibre and an associated stem (satellite) cell as a model. In response to tissue injury, local signals induce satellite cells to begin proliferating in order to generate sufficient progeny for tissue repair. Age-related changes in satellite cells, in the satellite-cell niche or in the systemic milieu could all result in a diminished functionality of satellite cells in an aged organism, manifested as a decreased propensity to generate sufficient functional progeny for effective regeneration.

Given the decline in satellite cell function with age, an important question regards the mechanisms underlying adult stem cell homeostasis. Age-related changes to adult skeletal muscle satellite cells are strongly influenced by environmental factors during ageing (Fig. **7**). This suggests satellite cell function may be reversible in the ageing muscle environment. This reversibility in satellite cell function was demonstrated in heterochronic parabiosis experiments whereby the circulatory system of old and young animals was joined [17, 70]. Satellite cells that were isolated from the skeletal muscle of ageing mice or humans failed to upregulate the NOTCH ligand DELTA. Furthermore, both showed increased signalling through the transforming growth factor (TGF)-ß pathway that led to defective satellite cell activation and proliferation following injury [71-73]. However, circulating factors from the blood of young mice restored NOTCH signalling and proliferative potential in satellite cells from old mice [17]. Proliferation defects of old human satellite cells can also be partially restored when cultured in the presence of NOTCH inhibitors [73]. Thus, it seems that the diminished regenerative potential of aged muscle is not primarily due to intrinsic ageing of satellite cells, but rather to the effects of the aged environment on satellite-cell function (Fig. **7**).

As mentioned epigenetic regulators may be suited as potential therapeutic targets to mediate changes in DNA methylation and histone modifications as they provide a versatile checkpoint for changes in gene expression and thus stimulating satellite cell activation and regeneration in ageing skeletal muscle. Other systemic factors that potentially regulate reversible stem cell function during ageing include cytokines, growth factors and stress hormones. For example, increasing the declining levels of the anabolic growth factor insulin growth factor-I (IGF-1) in aged rodents stimulates satellite cell dependent skeletal muscle regeneration [74, 75]. IGF-I has considerable anabolic effects in skeletal muscle, demonstrable when its expression is genetically manipulated [76]. Indeed signalling through the insulin/IGF-I pro-survival pathway, IGF-I, in particular, is a potent hypertrophic polypeptide and circulating IGF-I levels are an important determinant of skeletal muscle/myoblast function in the adult [77] and highlights the need to elucidate the potential mechanisms by external stimuli may induce epigenetic modifications in ageing muscle that lead to reversible changes in satellite cell function. In further support of reversible alterations to stem cell function in some tissues resulting from changes in external stimuli, such as physical exercise [78, 79], improve age-related declines in satellite cell function. Taken together, these observations suggest that a variety of environmental signals impact on intracellular mechanisms that can restore the potential of some adult stem cell types during ageing. Elucidating the mechanisms and potential epigenetic mechanisms that influence this reversible changes may provide novel targets for future drug therapy.

## FUTURE DIRECTIONS

**Fig. (8):**
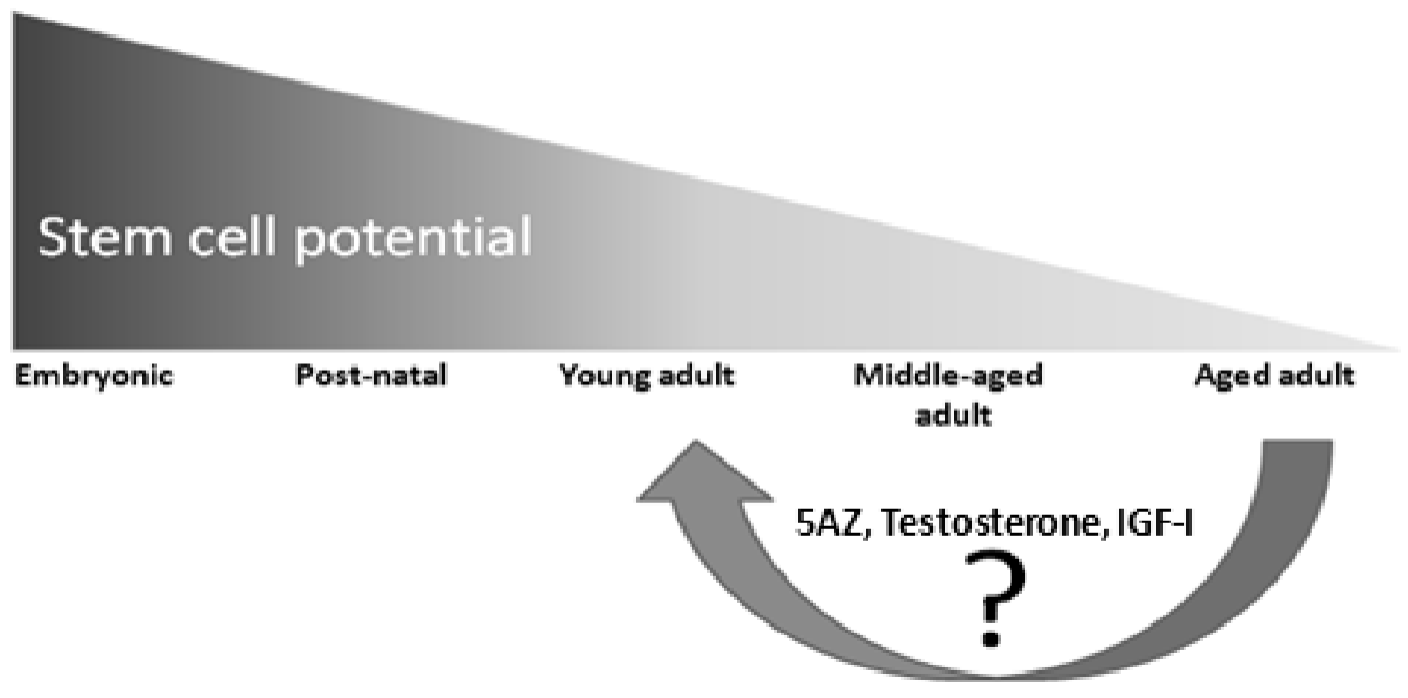
Satellite stem cell potential declines with age (adapted from [30]). During ageing, satellite stem cells lose their potential to regenerate tissues after damage because of decreased proliferation and differentiation potential. An important question is whether reversible epigenetic changes could underlie this decline in satellite stem cells. Chromatin modifiers and transcription factors may play an important role in restoring the regenerative capacity of old stem cells.

Whilst irreversible changes such as DNA associated with ageing inducing reversible changes, the potential for stem cells to be used as therapeutic vehicles has had a profound effect on the vision of the future of regenerative medicine (Fig. **8**). The suppression of adult stem-cell proliferation by the systemic milieu in aged animals, although it limits tissue regenerative potential and possibly promotes senescence or apoptosis, might be a crucial defence against the development of cancer, the likelihood of which increases with the accumulation of mutations in the stem cell genome with age. A greater depth of understanding of stem-cell biology, the environment of the aged tissue and the interaction between the two is essential if the therapeutic application of adult stem cells is to be realised in the context of regenerative medicine and ageing tissue repair.

Caution must be taken in delineating the role of chromatin regulators and transcription factors in stem cell maintenance and function as these have the capacity to induce mitosis of cancers and tumour development, as well as increased risks of cellular transformation. However, studies on the effect of exercise, environmental enrichment on old stem cells have revealed that stem cells rejuvenation can be achieved without the acquisition of neoplastic properties. By indentifying the synergistic and/or antagonistic interaction of DNA and chromatin regulators, their primary targets and the external signalling pathways that regulate these modifiers will be crucial to regulating the function of ageing stem cells and ensuring that regeneration is achieved in a controlled manner. Certainly, there is significant potential of utilising the regenerative capacity of dormant endogenous stem cells for both the prevention and treatment of age-related diseases associated with tissue degeneration. Application of this research has been made more promising given the recent ability to generate *in vitro* pluripotent stem cells from adult patients. The consequence of which has opened exciting new paths for exogenous stem cell therapies for the treatment of age-related diseases [30]. The area of health and ageing that is likely to benefit soonest from advances in the biology of adult stem cells is the emerging field of regenerative medicine. Possible application in regenerative medicine might be enhancement of endogenous stem cells or the transplantation of exogenous stem cells that have been expanded in culture. With regard to transplantation of exogenous stem cells into damaged tissues this will require firm foundations on the principles of transplantation biology and immunology. A greater appreciation for the importance of the aged environment of the tissue will be critical for the successful transplantation of stem cells in enhancing repair in the setting of acute injury. With regard to treatment of chronic regenerative diseases complexity of the host environment will require consideration for the use of stem cells in regeneration and repair. In the context of ageing, stem cell properties and the role of epigenetic pathways identified in animal models to stimulate tissue repair can be translational into humans will be critical steps in implementing new therapies.

## SUMMARY

The relationship between satellite stem cell function and muscle regeneration and repair in ageing has yet to be rigorously addressed in mammals. Initial *in vitro *studies of satellite cells from young and old animals suggested that there was intrinsic ageing of this stem-cell population, as aged cells generated far fewer progeny [80, 81]. The finding that regeneration mediated by aged satellite cells was highly effective when the cells were transplanted into young animals as whole-muscle grafts [82-84] suggest reversible modifications of aged muscle stem cells, an interpretation supported by recent data showing that aged muscle stem cells, when exposed to a youthful systemic milieu by virtue of parabiotic pairings of aged and young mice, activate and repair muscle nearly as well as young satellite cells [82]. These modifications may be associated with intrinsic epigenetic changes within the satellite stem cell population supported by our studies in myoblast cellular ageing demonstrating increased methylation of the myogenin gene in C2C12 myoblasts that reduced the capacity to form myotubes which was reversed by addition of methyltransferase inhibitor 5-azacytidine.

The coordinated control of DNA methylation by methyltransferases and chromatin states by histone modifiers will be a fascinating avenue for further investigation and will particularly benefit from genome-wide examination in young and old stem cell populations. As technologies for genomic analysis and sequencing continually improve profiling potential correlation between stem cell function and epigenetic changes become increasingly feasible. As the mechanisms underlying age dependent stem cell decline are better understood that leads to a decline in muscle function, studying the effects of manipulating satellite cell function on skeletal muscle maintenance over a organismal lifespan and healthspan will be an attainable goal – ‘from death, lead me to immortality’.

## Figures and Tables

**Table 1.** Potential Causes of Age-related Loss of Muscle Mass (Adapted From [2])

Increased sedentary lifestyle
Reduced levels of and responsiveness to trophic hormones
Reduced growth hormone levels
Reduced androgens (testosterone)
Decreased insulin-like growth factor-I and growth factor signalling
Lowered Dehydroepiandrosterone sulphate (DHEAS)
Decreased Estrogens (estrone, estradiol)
Decreased 25-hydroxy ergocalciferol (vitamin D)
Decrease or imbalance in protein metabolism
Increase in neurodegenerative process
Muscle fibre atrophy
Inflammation
Increased prevalence of disability
Decreased functional capacity
Decreased basal metabolic rate
Alteration in gene expression

**Table 2.** Mechanisms of Satellite Stem Cell Ageing: Irreversible Versus Reversible Changes [30]

Irreversible Changes	Reversible Changes

	Signalling pathways
DNA damage	Transcription factor activity
Telomere erosion	Chromatin State
Mitochondrial dysfunction	DNA methylation
